# Customizing supercontinuum generation via on-chip adaptive temporal pulse-splitting

**DOI:** 10.1038/s41467-018-07141-w

**Published:** 2018-11-20

**Authors:** Benjamin Wetzel, Michael Kues, Piotr Roztocki, Christian Reimer, Pierre-Luc Godin, Maxwell Rowley, Brent E. Little, Sai T. Chu, Evgeny A. Viktorov, David J. Moss, Alessia Pasquazi, Marco Peccianti, Roberto Morandotti

**Affiliations:** 1 Institut National de la Recherche Scientifique, Université du Québec, Varennes, QC J3X 1S2 Canada; 2 School of Mathematical and Physical Sciences, Department of Physics & Astronomy, University of Sussex, Falmer, Brighton, BN1 9QH UK; 3 School of Engineering, University of Glasgow, Rankine Building Oakfield Avenue, Glasgow, G12 8LT UK; 4 John A. Paulson School of Engineering and Applied Sciences, Harvard University, Cambridge, 02138 USA; 5 State Key Laboratory of Transient Optics and Photonics, Xi’an Institute of Optics and Precision Mechanics, Chinese Academy of Science, Xinxi Ave, Xi’an, Shaanxi China; 6 Department of Physics and Materials Science, City University of Hong Kong, Tat Chee Avenue, Kowloon, Hong Kong China; 7 ITMO University, 199034 St. Petersburg, Russia; 8 Centre for Micro-Photonics, Swinburne University of Technology, Hawthorn, VIC 3122 Australia; 9 Institute of Fundamental and Frontier Sciences, University of Electronic Science and Technology of China, Chengdu, 610054 Sichuan China

## Abstract

Modern optical systems increasingly rely on complex physical processes that require accessible control to meet target performance characteristics. In particular, advanced light sources, sought for, for example, imaging and metrology, are based on nonlinear optical dynamics whose output properties must often finely match application requirements. However, in these systems, the availability of control parameters (e.g., the optical field shape, as well as propagation medium properties) and the means to adjust them in a versatile manner are usually limited. Moreover, numerically finding the optimal parameter set for such complex dynamics is typically computationally intractable. Here, we use an actively controlled photonic chip to prepare and manipulate patterns of femtosecond optical pulses that give access to an enhanced parameter space in the framework of supercontinuum generation. Taking advantage of machine learning concepts, we exploit this tunable access and experimentally demonstrate the customization of nonlinear interactions for tailoring supercontinuum properties.

## Abstract (web summary)

Controlling complex properties of optical systems, like the output of nonlinear light sources, is increasingly important for applications. Here, Wetzel et al. use an actively-controlled photonic chip to prepare patterns of femtosecond laser pulses used for tailoring supercontinuum generation.

## Introduction

Complexity is a key characteristic of numerous physical systems, ranging from self-organisation to network access. Based on nonlinear or chaotic dynamics, and relying on a large number of parameters usually difficult to access, these complex systems have an ever-growing impact on our everyday life^1^. In photonics, numerous systems fall within this category where, for instance, the development of advanced optical sources^2–4^ is of tremendous importance for applications ranging from imaging to metrology^5–7^. A relevant example of this problem is the generation of a supercontinuum (SC)^8^, a broadband spectrum produced by an optical pulse propagating in a medium under the combined actions of dispersion, nonlinearities, and scattering effects^9,10^. In fibre-based systems, its underlying formation mechanisms are now well-understood and described within the framework of a modified nonlinear Schrödinger equation^3^. Significant work recently focused on studying broadening effects triggered by modulation instability processes^11^, initiated by long (i.e. sup-picosecond, >1 ps) pulses. In this case, the propagation dynamics are widely influenced by noise effects, ultimately resulting in incoherent output spectra^12,13^. However, many applications, including advanced metrology and imaging, today rely on coherent supercontinua^8^, where reproducible and controllable features are particularly required^6,7^. Specifically, for e.g. fluorescence imaging, pump-probe measurement techniques, spectroscopy, as well as coherence tomography, versatile control of both spectral and temporal SC properties is essential^7,14–17^. Yet, reproducible SC generation typically requires ultrashort (i.e. sub-picosecond, <1 ps) pulses^8^, where the means for controlling the propagation dynamics in a reconfigurable manner are limited^10,18^ (i.e. control is constrained by the design of the initial pulse condition and the properties of the propagating medium^19–21^). Therefore, in spite of numerous, rigorous studies of this regime, the optimization of coherent SC continues to be an experimental challenge^14,15,22,23^.

Specifically, the properties of single pulses deterministically seeding the generation of coherent supercontinua can be tuned over different degrees of freedom using state-of-the-art techniques (via e.g. pulse shaping, polarization control, or acousto/electro-optic modulation)^10,17,18,23–29^. However, these approaches typically rely on external devices that present fundamental limitations in the sub-picosecond regime, on top of being affected by complexity, bulkiness, and costs. More importantly, these techniques only allow for the control of very few single degrees of freedom (e.g. a single pulse’s duration, shape, or power), restricting the available nonlinear dynamics to intra-pulse effects such as soliton deterministic ejection and frequency shifts^3,30^, thus ultimately hampering the ability to optimize the overall system output.

In contrast, using more than one pulse to seed SC generation not only allows the excitation of multiple independent (intra-pulse) dynamics, but also leads to a richer variety of effects arising from the interaction of different components, i.e. inter-pulse effects such as multi-pulse soliton ejections, dispersive wave radiations, spectral superposition, steering, and collisions^3,30–34^. Yet, initial efforts towards this direction^32,33,35–38^ have only partially unleashed the potential of this concept. Recent approaches have been limited to the use of long (sup-picosecond) incoherent seeds, or to mutually-coherent pulses with very restricted control over their number and properties. Consequently, the full extent of SC control via multi-pulse seeding has yet to be achieved.

In this article, we demonstrate a method to drastically enhance the control parameter space for the tailoring of nonlinear interactions in guided fibre propagation and achieved SC generation with highly controllable properties. Using the length scales and stability available with integrated photonics^39^, custom sets of multiple and mutually-coherent ultrashort optical pulses (with low, 1 ps minimal separation) are prepared using optical pulse-splitting on a photonic chip, also enabling the adjustment of their individual properties (e.g. power, shape, chirp). The size of the control parameter space (i.e. over 10^36^ unique parameter combinations for 256 pulses) makes traditional experimental approaches, based on trial-and-error or exhaustive parameter sweeps, impossible. However, using machine learning concepts, in a similar fashion to approaches demonstrated in a variety of adaptive control scenarios^24,40–43^, we are able to optimize different pulse patterns and experimentally obtain the desired SC outputs. Specifically, we measure the spectral output and employ a genetic algorithm (GA)^44,45^ to modify the integrated pulse-splitter settings in order to optimize the nonlinear fibre propagation dynamics towards a selected SC criterion (for instance, maximizing the spectral intensity at one or more targeted wavelengths). The results of this proof-of-concept demonstration exhibit versatile control of the output spectra, allowing us to experimentally achieve a seven-fold increase of the targeted SC spectral density when compared to a single pulse excitation with the same power budget. Additionally, we numerically show the potential of this technique, not only for spectral shaping, but also towards the full temporal control of SC generation.

## Results

### Experimental setup

**Fig. 1:**
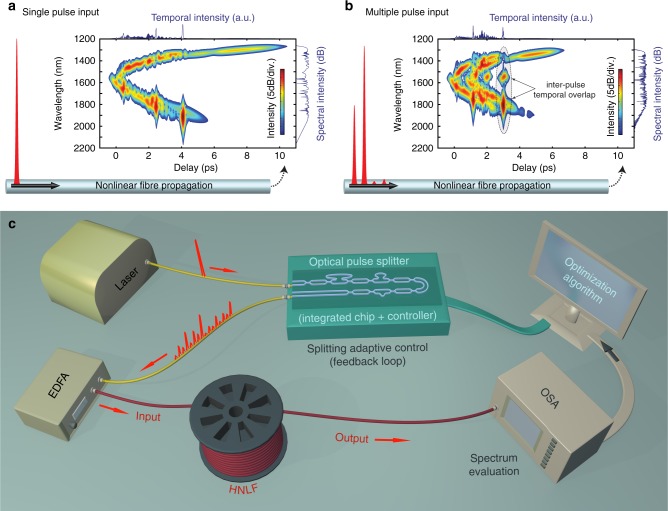
Concept of supercontinuum spectral customization via multiple pulse seeding. **a** Example of spectro-temporal properties (spectrogram^53^) of a single sub-picosecond pulse after propagation in a nonlinear optical fibre (see Methods). The newly-created spectral components experience progressive temporal walk-off^9,30^. At a given distance, only a few of the components temporally overlap, limiting nonlinear effects to intra-pulse interactions and restricting spectral shaping capabilities. **b** Using several pulses, more complex intra- and inter-pulse dynamics can be excited. Pulse-to-pulse separations on the order of the temporal walk-offs (~ps), enable the interplay of various spectral components generated from different pulses, thus providing enhanced nonlinear control over the spectral shaping. **c** Experimental setup: An integrated pulse-splitter is used to generate a custom train of pulses with ps-separation. After amplification with an erbium-doped fibre amplifier (EDFA), these are injected into a 1 km-long, highly-nonlinear fibre (HNLF) to form a supercontinuum, monitored using an optical spectrum analyser (OSA). A feedback loop is used to optimize the seed pulse train and tailor the supercontinuum output

**Fig. 2:**
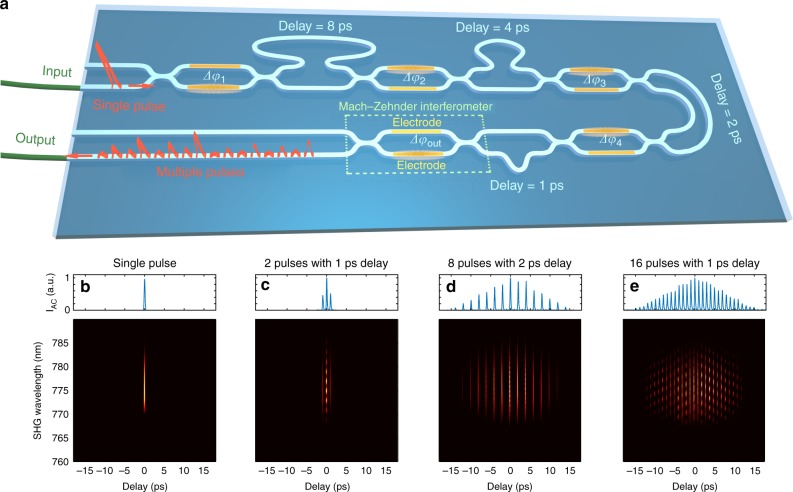
Operational principle of the on-chip optical pulse-splitter. **a** Schematic diagram: The sample comprises cascaded Mach-Zehnder interferometers (MZI) and different delay lines made of high-refractive-index silica glass (white)—see Methods for details. Adjustable splitting and routing of the optical pulses into the various waveguide paths can be controlled by tuning each MZI arm phase difference *Δφ*_*N*_ (where *N* is the interferometer number) via a refractive index change, thermally-induced using electrodes (yellow). We show in the figure a diagram comprising four interferometers used to adjust the relative splitting of pulses into the two arms of the subsequent unbalanced waveguide section. The last interferometer (*Δφ*_out_) is used to recombine the train of delayed pulses and regulate the overall output power. **b**–**e** Examples of generated optical pulse patterns characterized using intensity autocorrelation (AC—top) and frequency-resolved optical gating (FROG—bottom) measurements^53^. Multiple pulses (up to 16), featured by equal power but different pulse-to-pulse separation (as low as 1 ps), were obtained by setting each of the *N* = 4 interferometers to predefined splitting conditions, in order to sequentially split and interleave pulse trains with variable relative delays. These simple pulse pattern examples were achieved by setting each MZI relative phase difference to one of three specific values only: *Δφ*_*N*_ = 0, so that the incoming signal (i.e. pulse or train of pulses) entirely propagates within the short arm of the unbalanced waveguide section; *Δφ*_*N*_ = *π*, so that the incoming signal entirely propagates within the long arm of the unbalanced waveguide section; *Δφ*_*N*_ = ±π/2, so that the incoming signal is equally split between the two arms of the unbalanced waveguide section (leading to a relative delay corresponding to the unbalance between these two arms). Parameters: *Δφ*_1–4_ = 0 for (**b**); *Δφ*_1–3_ = 0 and *Δφ*_4_ = *π*/2 for (**c**); *Δφ*_1–3_ = *π*/2 and *Δφ*_4_ = 0 for (**d**); *Δφ*_1–4_ = *π*/2 for (**e**)

The approach proposed for the customization of nonlinear interactions via multiple pulse seeding is illustrated in Fig. 1. Our experimental setup (see Fig. 1c) comprises custom pulse train preparation via an integrated pulse-splitter and subsequent optical amplification, after which the multiple ultrashort pulses (~200 fs duration) were sent through 1 km of highly-nonlinear fibre (HNLF) in order to generate a SC. The spectrum of the pulse train measured at the HNLF input was centred around 1550 nm and had a 25 nm bandwidth (full-width at half maximum), corresponding to a 59 nm bandwidth at −10 dB. The input spectrum exhibited a small asymmetry (see Supplementary Fig. 1, 3 and Supplementary Discussion for details on the initial conditions) as well as an envelope modulation, whose properties depended on the pattern (i.e. number and separation) of the pulses generated by our integrated systems. Following fibre propagation and spectral broadening, the SC output was measured using an optical spectrum analyser and assessed with respect to target criteria (see Methods). The integrated device consists of a concatenation of balanced and unbalanced Mach-Zehnder interferometers (MZI), as illustrated in Fig. 2 (see Methods for details). The interferometers are electronically controlled via the use of integrated electrodes, which are responsible for thermally inducing an optical phase difference between the two arms of the interferometer. By adjusting the interferometers’ splitting ratio, an input femtosecond pulse is divided into multiple fractions. Those will follow different path combinations within the waveguide structure of the CMOS-compatible photonic chip, allowing the preparation of a coherent train of multiple pulses with adjustable peak powers and relative delays (with as low as 1 ps pulse separation, see Fig. 2b–e). The device exhibits low losses (~3 dB overall, see Methods) and the interferometers’ integrated push-pull configuration provides excellent repeatability and stability against environmental perturbations. More importantly, the photonic chip enables versatile control of the pulse train (i.e. power, delay, pulse duration, chirp, etc.): Specifically, the large Kerr nonlinearity and weak anomalous dispersion in the device waveguides^46^ can bring about path- and power-dependent nonlinear phase shifts and temporal broadening. These control properties are very important for the optimization of coherent SC features: the ability to adjust multiple pulse shapes, chirps, powers, as well as their relative delays and phases constitutes the key ingredient required for the efficient control of independent and variable deterministic soliton radiation processes (i.e. intra-pulse dynamics) at the basis of spectral broadening in the current propagation regime. This control of the initial parameter space (and the corresponding intra-pulse dynamics leading to subsequent soliton radiation) is also expected to condition inter-pulse dynamics during further fibre propagation, including the tuning of multiple soliton interactions such as repulsion, collision, or spectral superposition^8,9,30–33^. The use of multiple yet coherent pulse excitations is thus foreseen as a simple way to customize a wide variety of nonlinear interactions which are otherwise hard to tune using conventional pulse shaping techniques. Remarkably, they are here accessible in a simple yet efficient integrated platform.

### Single-wavelength optimization

**Fig. 3:**
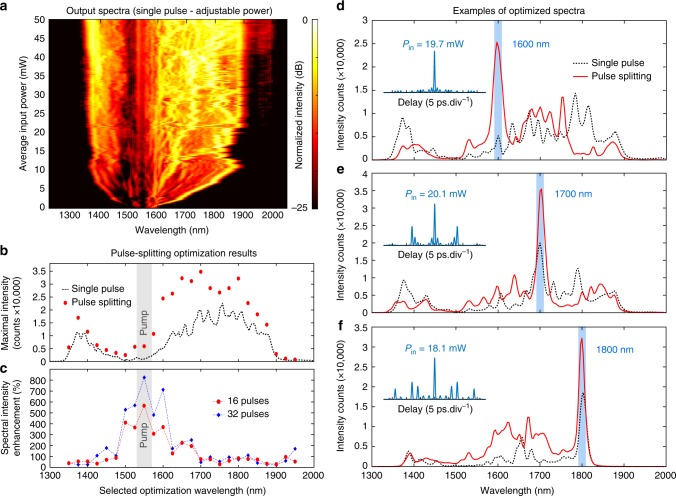
Supercontinuum spectral intensity optimization at selected wavelengths. **a** Spectral intensity map measured at the highly-nonlinear fibre output, generated by a single pulse seed as a function of its average power. **b** Maximal spectral intensity reached as a function of the selected optimization wavelength, considering either a single pulse seed case (dashed black line—i.e. the maximal intensity retrieved from panel **a**), or using the pulse-splitting optimization technique (with up to 16 pulses—red circles) for the same power budget—see Methods. **c** Spectral intensity enhancement (relative to the single pulse seed case as a reference), for pulse-splitting performed with 16 (red dots) or 32 (blue diamonds) seed pulses. For reference, the input pump spectral location is shown as grey shadings in (**b**) and (**c**). **d**–**f** Examples of spectra obtained following intensity maximization at target wavelengths (blue shadings), using single pulse seeding (dashed black lines), or pulse-splitting optimization (red lines—with up to 16 pulses). The insets show the autocorrelation traces of the corresponding, optimal input pulse trains and average powers *P*_in_

In order to illustrate the versatility of our scheme for controlling nonlinear pulse propagation, we first demonstrated the enhancement of the power density at a single wavelength of the SC. For this, we limit our study to two cases. As a reference, we used the simplified case of a SC spectrum generated by a single pulse with adjustable power (Fig. 3a). Here, spectral broadening was mediated by the radiation of multiple solitons and dispersive waves, which subsequently experienced Raman self-frequency shifts^9,30^. For comparison, our second case used multiple excitation pulses (prepared using the integrated splitter), possessing the same power budget as in the single pulse study (50 mW), but with input parameters refined via genetic algorithm optimization (see Methods).

We found that, for target wavelengths across the SC bandwidth, the use of multiple pulses enabled 20–700% spectral density enhancement relative to the reference (see Fig. 3b, c). Examples of optimized spectra for three particular target wavelengths are shown in Fig. 3d–f and illustrate how the resulting spectra can vary significantly for a similar input power but drastically different pulse configurations (see insets). Note that, in this work, we restricted our study to a limited number of pulse seeds (either 16 or 32 pulses in the pattern instead of the 256 maximally-achievable with the chip). Nevertheless, in the propagation regime studied (where SC spectral broadening above 2000 nm is intrinsically limited by fibre losses), the use of such a subset of the available parameter space is sufficient to exhibit notable spectral enhancement while ensuring fast convergence in the optimization process (see Methods and Supplementary Fig. 4, for discussion). As expected, the use of 32 pulse seeds was found to outperform the use of 16 pulse seeds (see Fig. 3c). In this regime, such expected behaviour can be explained by the potential of multiple pulse seeds to judiciously condition the spectral steering and, ultimately, the superposition of independently generated spectral components (see Supplementary Fig. 2). It is foreseen that for other applications and target SC outputs, a greater number of pulses, and consequently larger parameter spaces, will enable even better performances.

### Dual-wavelength optimization

**Fig. 4:**
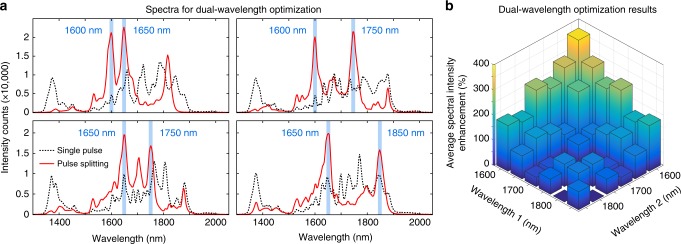
Supercontinuum spectral intensity optimization for different wavelength pair combinations. **a** Examples of spectra obtained following simultaneous intensity maximization at two target wavelengths (blue shadings), using single pulse seeding (dashed black lines), or pulse-splitting optimizations (red lines—with up to 16 pulses). Note that we used here the same setup and power budget as in Fig. 3, and simply modified the algorithm optimization criteria (see Methods). **b** Optimization matrix for wavelength pair combinations, showing normalized intensity enhancement obtained for combinations of wavelength pairs. Such enhancement (see colour bar on the left axis) is calculated as the average intensity at both wavelengths and is normalized relatively to the single pulse seeding case (see Methods). For clarity, we only report results where the intensity at one wavelength is less than twice as large as the intensity at the other wavelength (see Supplementary Fig. 5, for a complete analysis)

Remarkably, the ability to generate multiple pulses with specific delays and properties further enables the control and optimization of typically complex and interdependent dynamics. This feature is illustrated in Fig. 4, where simultaneous enhancement of the power density at two distinct SC wavelengths can be obtained with our scheme (see Methods). Here, we specifically targeted cases where both wavelength intensities were equivalent (see Methods), but further tunability is accessible depending on the exact optimization criteria used for the algorithm (see Supplementary Discussion and Supplementary Fig. 5). Indeed, arbitrary optimization criteria can be implemented^44^, in stark contrast to what can be obtained with a single pulse seed. Spectral broadening mediated by soliton radiation is highly deterministic and typically leads to strong spectral correlation in the resulting SC^3,31,32,47^. In our case however, multiple pulse excitation can seed both independent dynamics and customized nonlinear interaction. This, alone, manifests a powerful example of how our integrated system, along with the implementation of machine learning concepts, can be efficiently used to tailor complex nonlinear processes without extensive system design.

### Advanced spectro-temporal control

**Fig. 5:**
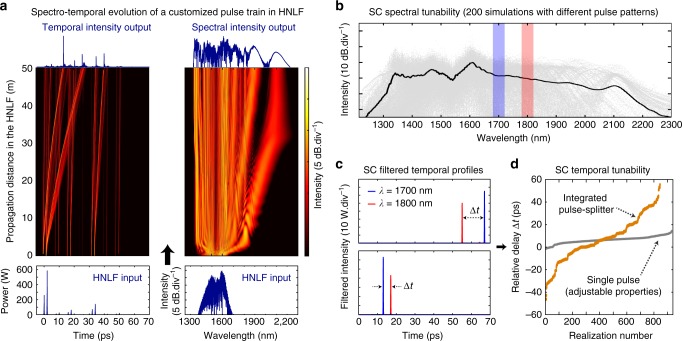
Numerical simulations showing control of the supercontinuum spectral and temporal properties. **a** Example of supercontinuum (SC) temporal (left) and spectral (right) evolution in 50 m of highly-nonlinear fibre (HNLF). A train of 64 pulses, prepared using the integrated pulse-splitter (bottom), is injected into the HNLF to generate a broadband supercontinuum (top). **b** SC spectra obtained by simulating the propagation of 200 randomly prepared pulse patterns (grey). The average spectrum of these is plotted in black. Additional numerical analysis shows that despite different evolution dynamics, the respective spectra individually retain a high average degree of coherence <*g*> thanks to the (coherent) optical splitting method employed (The average coherence of each individual spectrum illustrated, computed over a 20 dB bandwidth, was <*g*> = 0.973—see Methods)^3,7,39^. **c** Examples of two different SC temporal profiles (top and bottom panel—obtained from two different input pulse patterns) after narrowband filtering at two specific wavelengths (i.e. 1700 and 1800 nm—see blue and red shadings in (**b**), respectively), showing that the differently-coloured pulses can exhibit diverse arrival times. **d** The relative delay between these filtered output pulses is computed for various integrated pulse-splitter configurations (brown dots). We found an enhanced temporal tunability compared to SC generated from a single input pulse with randomly adjusted properties (grey squares)—see Methods

Additionally, our approach has the potential of controlling the SC temporal properties, which we confirmed by numerical simulations using a shorter fibre propagation length (in order to ensure reliable and reasonably fast computation of the pulse propagation dynamics—see Methods for details). In particular, by assuming a 200 fs pulse (2 kW peak power), which was randomly split into a train of 64 pulses by our integrated device and propagated through a 50 m-long HNLF, we show how solitons, radiating from the excitation pulses, can individually experience different Raman self-frequency shifts and temporal walk-offs leading to collisions, steering and the generation of novel frequency components^14,32,33^ (see Fig. 5a). Overall, such an enhanced parameter space can ultimately drive different propagation scenarios and thus provide a high degree of reconfigurability in terms of SC properties (see Supplementary Fig. 2 for different examples of propagation dynamics). Indeed, depending on the initial conditions, highly variable yet coherent SC output spectra^3^ can be obtained (Fig. 5b), with the additional possibility for selecting the delay and order with which specific spectral components emerge from the fibre (see Fig. 5c)^14^.

Continuous control of the relative delay between two spectral components can thus be obtained over a large temporal window of ±40 ps (Fig. 5d—see Methods). This ability, obtained by exploiting the multiple pulses of the system, provides a higher flexibility and tuning range with respect to conventional shaping techniques applied to a single pulse (see Fig. 5d and Methods for details)^10,17,18,22,26,28^. In particular, when considering the propagation regime studied here, single pulse seeding always leads to the formation of soliton(s) and associated dispersive waves, the temporal walk-off of which is intrinsically related to their wavelength (i.e. the most red-shifted solitons will typically exhibit a larger delay due to Raman scattering^8,9^). In this framework, even conventional shaping techniques are inherently limited to slightly adjusting the absolute value of the relative delays between different spectral components (see Fig. 5d). On the other hand, the use of multiple pulse seeding has shown that versatile temporal control between two (or eventually more) pulses at arbitrary wavelengths of the output spectrum can be obtained. This additional temporal tunability, typically required e.g. in advanced imaging systems^14,16,17^, will complement the experimentally-demonstrated spectral shaping, and is expected to be further enhanced (e.g., by activating additional interferometers and thus exponentially expanding the accessible parameter space).

## Discussion

We demonstrate how adjustable, integrated path-routing can be used to access a wide and controllable optical parameter space. In combination with the use of genetic algorithms (GAs), we showed the generation of supercontinua with broadly reconfigurable characteristics. Most importantly, this is achieved with the same power budget, meaning no additional amplification was used, and therefore the benefits of pulse splitting far exceed the drawbacks due to the additional optical loss of the integrated device. In particular, the improvements provided by the additional degrees of freedom in the multi-pulse excitation regime can condition the interleaving, superposition, and nonlinear interaction between multiple phase-locked pulses, which in turn significantly expands the controllable SC properties by allowing the customization of both their spectral and temporal power distribution. Besides this demonstration in the telecom range, our approach could be extended to other typical laser wavelengths (e.g. 800 or 1064 nm) and/or fibre designs. Using for instance an optical source to seed a fibre with a judiciously chosen dispersion profile (in order to circumvent the loss-induced spectral broadening limitations observed in the current HNLF), coherent and reconfigurable octave spanning SC generation is expected to be readily obtained with our proposed systems. Similarly, the nonlinear fibre used in our experiments for SC generation could be shortened or readily integrated on a photonic chip^46,48,49^, thus providing a compact and stable system for the deployment of advanced optical functionalities (such as on-chip *f*-2*f* interferometry based on coherent SC^50^). In this context, we foresee this approach as an invaluable tool for the development of novel optical sources for, e.g. state-of-the-art imaging and metrology applications requiring both spectral and temporal tunability (e.g. pump-probe measurement techniques, hyperspectral imaging, or various schemes for coherent control)^4–8,14,16,34^.

Additionally, it is worth underlining that we restricted our attention to the use of sub-picosecond pulses (<1 ps) in order to avoid temporal overlap between adjacent excitation pulses during propagation in our integrated system. Yet, the design of a pulse-splitter with shorter relative delays or, equivalently, the use of longer pulses is also expected to unlock novel features in SC adaptive control (via e.g. coherent temporal synthesis^39^ and tailored pulse superposition^31,33^), especially within the framework of fundamental studies associated with extreme event formation^11,13,35,38^. Similarly, such an approach is thus expected to allow the optimal exploitation of complex optical systems without a priori knowledge of their dynamics. This may include applications related to advanced nonlinear signal processing^51^, the control of frequency comb emission^4,5,22^, as well as of laser mode-locking^2^. In turn, this can pave the way towards the next generation of self-adjusting lasers^40,52^ and ‘smart’ integrated optical systems.

## Methods

### Integrated pulse-splitter

The on-chip photonic pulse-splitter was formed by cascading *N* = 9 balanced interferometers and *M* = 8 delay lines based on integrated optical waveguides (see Fig. 2), where the associated delays varied according to the relation *Δτ*_*M*_ = 2*Δτ*_*M*-1_. The device was fabricated from a CMOS-compatible, high-refractive index silica glass (Hydex) produced by chemical vapour deposition without the need for high-temperature annealing^46^. Patterning was done using UV photolithography and reactive ion etching. This material platform is featured by a refractive index of *n* = 1.7, along with very low linear (0.06 dB cm^–1^) and negligible nonlinear optical losses (no nonlinear losses measured up to 25 GW cm^–2^). The waveguide dimensions allowed for single mode TE and TM propagation at telecom wavelengths, where the dispersion and nonlinear properties were similar to those reported in Ref.^46^, as verified by means of optical vector analyser measurements (Luna OVA 5000). At the central wavelength of the pulses used in our experiments (i.e. *λ* = 1550 nm) and for the selected polarization, we estimated the waveguide dispersion to be extremely low (*β*_*2*_ = −2.87 ps^2^ km^−1^and *β*_*3*_ = −0.0224 ps^3^ km^−1^), yet the effective nonlinearity to be significantly high (*γ* = 233 W^−1^ km^−1^).

The input and output bus waveguides featured mode converters and were pigtailed to 1.2 m fibre patchcords (SMF-28) on each side, resulting in coupling losses of 1.4 dB per facet. The total propagation length in the sample varies between 5 and 9.5 cm and depends on the selected paths inside the structure. Overall, the total losses in our pulse-splitter were measured to be between 3.10 and 3.36 dB at 1550 nm, depending on the optical path (including the attenuation from the mode converters and pigtails).

Gold electrodes were deposited on each arm of the nine balanced interferometers (the last is dedicated to the output splitting and pulse recombination but does not introduce any delay), in order to induce a local and variable thermal modification on the adjacent optical waveguide. Such a modification was controlled electronically (see below) to produce a phase difference *Δφ*_*N*_ between the two arms of the interferometer (where *N* is the interferometer number in the sample). For each of the eight interferometers, the waveguide thermal control allowed for a tunable switching of the optical output beam path between the short and the long arm of the following delay line (thus inducing a path difference equivalent to a delay *Δτ*_*M*_). The two output waveguides from such elements were then fed into the two input waveguides of the subsequent balanced interferometer. Via these cascaded blocks, it is possible to generate up to 2^*M*^ optical pulses with adjustable powers and temporal separation (multiples of *Δτ*_1_). We note that in our integrated pulse-splitter, the minimal delay was *Δτ*_1_ = 1 ps, and the maximal delay was *Δτ*_8_ = 128 ps so that we were able to generate up to 256 pulse replicas with adjustable individual powers and tunable temporal separation (multiples of 1 ps) over the range 1–255 ps. We also note that the photonic chip supports bidirectional optical propagation and can also be used by inverting the input and output ports, as illustrated in Fig. 2a (thus leading to a propagation where the associated delays in the chip decrease according to the relation *Δτ*_*M*_ = 0.5 *Δτ*_*M-*1_).

The splitting ratio of the interferometers was computer controlled, via a push-pull architecture implemented by custom computer-controlled driving electronics, used to apply up to the 5.0 ± 0.2 V voltage swing required to completely switch the beam path from one interferometer output to the other. The splitting ratio characterization was carried out by measuring the optical power at each interferometer and we found, for each element, an extinction ratio above 16 dB (i.e. a cross-talk below 2.5%). From both electronic and optical characterizations, we estimated that the splitting ratio can be modified with a resolution of 0.01% (assuming a wavelength independent response) via ~32,000 voltage control levels per interferometer over the voltage range required for a complete path switching. For the overall sample, this corresponds to more than 10^36^ different setting configurations (i.e. combinations) that can be employed for the versatile generation of multiple pulse replicas. The wavelength dependence of the interferometer splitting ratio was characterized using a tunable CW laser (Tunics-Plus). Over the range 1525–1575 nm (i.e. the bandwidth of the pulses used in our experiments), we found only negligible differences in the measured splitting ratio (i.e. maximal ± 3%) and overall losses (~0.2 dB discrepancies in the worst-case scenario, when propagating along a single waveguide path).

Finally, the response time of the system was estimated by switching one (or several) interferometer splitting ratios within the sample, and temporally resolving the subsequent SC spectral modification induced after propagation in the HNLF. This was done using a fast spectrometer (see Methods below), which was also used to ensure the long-term stability and excellent repeatability of the pulse-splitter device. For one interferometer, the switching settling time (to reach thermal equilibrium) for the maximal voltage swing, was estimated to be below 100 ms, including the overall lag time of the computer, driving electronics, and detection system (~30 ms overall). Using simultaneous switching of five interferometers, we observed slightly longer settling times that were attributed to thermal cross-talk between adjacent interferometers, as well as longer update times of the sequential commands sent to the electronic drivers (~160 ms). An overall 500 ms settling time was estimated to be sufficient for capturing the main spectral modifications in the experiments performed here while ensuring excellent repeatability. This was confirmed, in case of the optimization routine, by choosing an extremely conservative 3 s settling time, yielding equivalent results to those obtained using a 500 ms settling time.

### Experimental SC generation and control

The fibre laser used in our experiments (Menlo C-Fiber) generated femtosecond pulses at a 250 MHz repetition rate. The initial spectrum, measured with an optical spectrum analyser (OSA), was centred at 1550 nm with a 52 nm bandwidth (full width at half maximum—FWHM). After suitable dispersion management and temporal recompression, the pulse was sent into our integrated pulse-splitter, allowing for the preparation of multiple pulses. The optical pulse had an estimated ~ 200 fs duration (FWHM) and peak power of 300 W when entering the photonic sample. Subsequently, the optical output of the sample (i.e. the set of prepared sub-picosecond pulses) was amplified to the desired power level by using a short length (1.6 m) erbium-doped fibre amplifier (EDFA) before being injected into 1 km of HNLF. At this wavelength, the fibre operates in the anomalous dispersion regime, yet close to the zero-dispersion wavelength (ZDW = 1545 nm, see HNLF parameters below). After propagation in the HNLF, the broadband SC was characterized using a fast spectrum analyser (Avantes—AvaSpec NIR512) allowing measurements over a 954–2580 nm window with a resolution of ~3.5 nm. The spectral intensity(ies) at the wavelength(s) of interest were extracted from these measurements for each iteration and then used to implement the optimization criterion for the GA (see Methods below and Supplementary Discussion). The measurements of the fast spectrum analyser were also performed over the range 600–1750 nm using the OSA (ANDO AQ6317B), thus allowing for consistency verification with improved resolution. Measurements of the initial conditions were carried out at the input of the HNLF: Pulse spectral and temporal diagnostics were done with the OSA as well as with a custom intensity autocorrelator and frequency-resolved optical gating setup (FROG)^53^.

Temporal broadening of the sub-picosecond optical pulses was controlled via careful dispersion management throughout the setup until injection into the HNLF. Specifically, we used a combination of single mode fibre (SMF-28) and dispersion compensating fibre (DCF-38 from Thorlabs) with patchcords of specifically chosen lengths to (i) minimize the initial chirp of the pulse emitted from the fibre laser, (ii) temporally recompress the pulse (while limiting nonlinear effects) before injection into the integrated splitter, as well as, (iii) maintain the pulse temporal spreading to a minimum during and after propagation in the sample. This configuration, based on controlled dispersion management and subsequent amplification after pulse-splitting, allows obtaining ~ 200 fs pulses both at the HNLF input and throughout propagation in the photonic chip waveguides (thus avoiding temporal overlap between the adjacent prepared pulses) while limiting the overall distortions induced via nonlinear effects before injection into the HNLF.

For the reference experiment using only a single pulse, the pulse-splitter was set with all splitting ratios to their minimal values (so that only one pulse follows the shortest possible waveguide path), while the EDFA current was tuned to linearly sweep the average power with 425 incremental steps over the range 0.1–50 mW. Note that we used an additional optical attenuator before the HNLF for measuring SC output spectra with input average powers below 2.5 mW (i.e. the EDFA amplification threshold). The best result within this ensemble of 425 output spectra—with respect to the desired optimization criterion—was used as reference for comparing the results obtained via multiple pulse optimization.

For these experiments, we set the EDFA current to the maximal value used previously (i.e. leading to 50 mW average output power after amplification). In this case, the relative powers of the pulses were directly controlled by modifying each delay interferometer splitting ratio (as well as the output interferometer, for regulating the overall power).

### GA parameters

For the experiments presented, we made use of an optimization process based on a GA implemented directly from the Matlab dedicated function which was employed to adjust the integrated pulse-splitter parameters via driving electronics controlled through a USB connection. Note that no other tuning parameters were used and all other experimental settings were kept constant. For assessing the optimization criteria, we used the spectral intensity measured at one (or two) wavelength(s) in the SC. For the reported experiments, the GA was configured to enhance the discrete spectral intensity at a selected wavelength (i.e. single-objective function—as seen in Fig. 3) or both spectral intensities at two selected wavelengths (i.e. multiple-objective function—as seen in Fig. 4). In the latter case, the optimization process yields a so-called Pareto front (or frontier)^44^, corresponding to the optimum set of parameters leading to the best trade-offs between the optimization criteria. Note that in Fig. 4, we only illustrate the case where both spectral intensities at the selected wavelength of interest are similar. A detailed analysis of the Pareto front clearly indicates that more sophisticated optimization processes can readily be achieved (e.g. allowing to select the ratio between the spectral intensity at the desired wavelengths—see Supplementary Fig. 5 for additional details). This, in turn, leads to more versatile output properties.

For proof of principle demonstrations, we kept the parameters of the GA function to their default values and selected a crossover of 50%, i.e. the ratio of ‘genes’ (the voltage of a single interferometer) from each ‘individual’ (set of all voltages for the interferometer array) carried from one ‘generation’ to the next^44,45^. The number of individuals populating each generation (i.e. each iteration step of the GA) was adjusted depending on the number of genes for each individual (i.e. the number of actively-controlled interferometers).

In addition, the maximal number of generations was limited in order to achieve a meaningful optimization towards the targeted SC output in a reasonable time frame (~1 h for each optimization process). Specifically, for five active interferometers (i.e. for generating 16 pulses), we constrained our algorithm to 15 generations with 500 individuals. Correspondingly, when 6 interferometers were actively adjusted (i.e. for 32 pulses), we expanded the population size to 1,000 individuals in order to obtain a better sampling of the initial parameter space (i.e. one additional gene per individual), while reducing the number of generations to 10. Note that, with such a limited number of generations, a systematic convergence (in the strict mathematical definition) of the GA might not be fully reached. However, we obtained a consistent improvement in the desired SC properties even using this limited number of iterations. Such optimization has been carefully verified for various sets of GA parameters (as well as for various settling times—i.e. the time between setting the system and taking the measurement, see Supplementary Fig. 4 and Supplementary Discussion), and was also observed in additional tests for more complex optimization objectives.

### Numerical simulations of nonlinear pulse propagation

Our numerical simulations used a split-step Fourier method to solve the generalized nonlinear Schrödinger equation (GNLSE)^3,8^ for modelling the pulse evolution in both the on-chip photonic pulse-splitter and the HNLF:

1 $${\frac{{\partial A}}{{\partial z}} + \frac{\alpha }{2}A - \mathop {\sum}\limits_{k \ge 2} {\frac{{i^{k + 1}}}{{k!}}} \beta _k\frac{{\partial ^kA}}{{\partial T^k}} = i\gamma \left( {1 + i\tau _{{\mathrm{shock}}}\frac{\partial }{{\partial T}}} \right)\left( {A\left( {z,T} \right){\int}_{ - \infty }^{ + \infty } {R\left( {T\prime } \right)} \left| {A\left( {z,T - T\prime } \right)} \right|^2\mathrm{d}T\prime } \right)}$$

Here *A*(*z*, *T*) is the pulse envelope (in W^−1/2^), evolving in a comoving frame at the envelope group velocity *β*_1_^−1^, so that *T* = *t* − *β*_1_*z*. The model includes higher-order dispersion (shown on the left-hand side of the equation) and nonlinearity (on the right-hand side), as well as the presence of loss *α* and initial broadband noise (i.e. one photon with random phase per spectral mode)^9,10^. The overall nonlinearity is represented by a nonlinear coefficient *γ* and includes the self-steepening effect (through a shock timescale *τ*_shock_ = 1/*ω*_0_ = 0.823 fs). The nonlinear response function *R*(*T*′) = (1−*f*_R_)δ(*T*′) + *f*_R_*h*_R_(*T*′) encompasses both an instantaneous Kerr effect and a delayed Raman contribution *h*_R_(*T*′), the weight of which is given by *f*_R_ = 0.18.

For the HNLF, we used the parameters retrieved from the manufacturer datasheet (OFS Fitel—HNLF ZDW1546): At a central wavelength of 1550 nm, the nonlinear parameter is *γ* = 11.3 W^−1^ km^−1^, the dispersion is slightly anomalous with *β*_2_ = −0.102 ps^2^ km^−1^, *β*_3_ = 0.0278 ps^3^ km^−1^ and *β*_4_ = 4.0 × 10^−5^ ps^4^ km^−1^ and the linear losses are 0.99 dB km^−1^.

For simulations of the on-chip photonic pulse-splitter, we modelled the pulse evolution through each waveguide element using a split-step method. This included the dual waveguide system with balanced and unbalanced interferometric structures and associated beam splitter transfer functions. The splitting ratio of the MZI structures, allowing for tuning the pulse path and creating delayed pulse replicas, was modelled by adding a tunable phase offset on one arm of the respective balanced interferometer. The parameters of the waveguides at 1550 nm, assuming a pure TM polarization, were taken as *β*_2_ = −2.87 ps^2^ km^−1^ and *β*_3_ = −0.0224 ps^3^ km^−1^, with a nonlinear parameter *γ* = 233 W^−1^ km^−1^ and linear losses of 0.06 dB cm^–1^ (see above and ref. ^46^).

For the cases shown in Fig. 1, we simulated the evolution of a transform-limited Gaussian pulse of 200 fs duration (FWHM) with a peak power of 1 kW directly injected into 10 m of HNLF with the properties shown above (Fig. 1a), or split into four pulses with different peak powers and 1 ps separation (Fig. 1b). Note that the same individual properties and overall input energy was used for both cases. The corresponding spectrograms were constructed using a 50 fs hyperbolic-secant gate function^10,53^.

For the proof-of-concept simulations illustrated in Fig. 5, we considered a simple case where the integrated pulse-splitter was directly connected to 50 m of HNLF, with coupling losses of 1.4 dB per chip facet. At the input of the pulse-splitter, we injected a transform-limited Gaussian pulse of 200 fs duration (FWHM) with a peak power of 2 kW (i.e. the typical values associated with a fibre laser producing such pulses at 10 MHz repetition rate and 4 mW average output power). For this analysis, we carried out 10,000 simulations randomly varying the splitting ratio of 6 (+1 output) interferometers (such as to produce 64 pulses with 1 ps separation and adjustable powers). The extraction of each soliton/pulse properties at the HNLF output (see Fig. 5c) was carried out using a spectral filter with a 50 nm bandwidth (FWHM) around the wavelengths of interest. Subsequent analysis of the pulse relative delay at the HNLF output (see Fig. 5d) was obtained by discarding the filtered pulses with a peak power below 20 W (i.e. below 1% of the initial pulse peak power). For this diagram, we post-selected cases where only one pulse was obtained at each wavelength (after filtering) within the ensemble of 10,000 simulations.

The coherence analysis of the supercontinua was carried out by fixing the splitting ratio used in our model and performing 20 stochastic numerical simulations with random noise seeds (i.e. adding one photon with random phase per spectral mode). The degree of spectral coherence |*g*^(1)^_12_ (*λ*, 0)| was thus retrieved as the mean value for the modulus of the degree of first-order coherence calculated at each wavelength *λ* over this ensemble of 20 simulations, which we found to be a sufficient number for a meaningful estimation of the SC coherence^36^. <*g*> was computed as the mean spectral coherence over the 20 dB SC spectral bandwidth. Note that the coherence value mentioned in Fig. 5 was then obtained by repeating this procedure for 50 different pulse-splitter settings and then averaging the results.

Compared to a single input pulse with adjustable properties, the use of multiple and controllable pulses leads to enhanced tunability in the SC properties, which is illustrated in Fig. 5d. For this comparison, we repeated the previous set of 10,000 stochastic simulations replacing our integrated pulse-splitter by an arbitrary pulse shaper^25^. In particular, we modelled the random variation of the pulse properties by first modifying its peak power *P*_0_ and temporal profile asymmetry *ε*. The pulse envelope *A*(*T*) was constructed from two half-Gaussians with different widths being respectively $T_{\mathrm{0}}^ \pm = \left( {1 \pm \varepsilon } \right)T_{\mathrm{0}}$. These half-Gaussians were added so that their maxima overlap, forming a pulse of duration *T*_0_ = 200 fs (FWHM) with variable trailing/leading edge steepness. We then added a random quadratic and cubic spectral phase of the form $\exp \left( {i\eta \nu ^2 + i\kappa \nu ^3} \right)$ on top of the pulse spectrum $\tilde A(\nu )$, before finally implementing an additional nonlinear phase shift, i.e. a self-phase modulation with random and tunable nonlinearity of the form ${\mathrm{exp}}\left( {i\varphi _{{\mathrm{NL}}}\left| A \right|^2/P_{\mathrm{0}}} \right)$. Each of these parameters was randomly changed for 10,000 different realizations, and uniformly distributed over the ranges:*P*_0_ = [0 2] kW; *ε* = [−0.5 0.5]; *η* = [−0.5 0.5] ps^2^; *κ* = [−0.05 0.05] ps^3^; *φ*_NL_ = [−3*π* 3*π*]. Although not exhaustive, such adjustable properties are typical of common optical processing systems (e.g. extra fibre length, pulse spectral shaping, etc.) and allow for a direct comparison with our pulse-splitter-based simulations, as the typical SC output bandwidths and coherence degrees remained quantitatively similar for such pulse durations^3^. Note that the SC filtering, processing, and post-selection described above remained otherwise unchanged.

In order to verify the overall validity of the dynamics observed in our experiments, we also performed simulations of the evolution of a single pulse directly injected into 1 km of HNLF with variable input powers (see Supplementary Fig. 1). In this case, numerical simulations were carried out for propagation in the HNLF only, using the input conditions measured at the HNLF input from spectral (OSA) and temporal (autocorrelator/FROG) experimental characterization.

## Electronic supplementary material

Supplementary Information

## Data Availability

The data that support the findings of this study are available from the corresponding authors upon reasonable request.
