# Supplementary material for: Customizing supercontinuum generation via on-chip adaptive temporal pulse-splitting
Source: Nat Commun. 2018 Nov 20;9:4884. doi: 10.1038/s41467-018-07141-w (PMC6244003; doi:10.1038/s41467-018-07141-w)
Supplement: Supplementary file 1 — Supplementary Information [file 41467_2018_7141_MOESM1_ESM.pdf]

SUPPLEMENTARY INFORMATION

Customizing supercontinuum generation via on-chip adaptive temporal pulse-splitting

Wetzel *et al.*

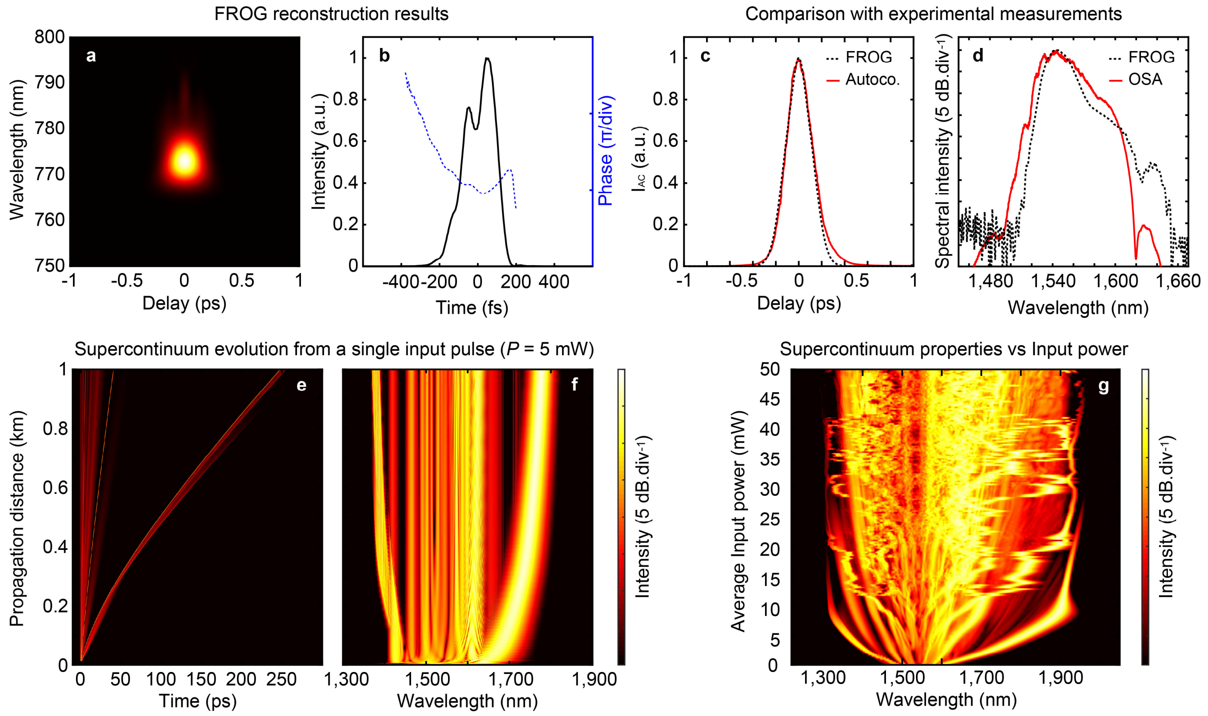

Supplementary Figure 1: Numerical simulation of supercontinuum evolution dynamics. (a) Reconstructed frequency-resolved optical gating (FROG) trace of the (single) optical pulse injected into the highly nonlinear fibre (HNLF) at the maximum amplification of the erbium-doped fibre amplifier (EDFA). (b) Corresponding pulse temporal properties retrieved from FROG reconstruction[1]. The intensity profile (left axis) is shown in black and the temporal phase (right axis) is depicted as a dashed blue line. (c) The autocorrelation trace retrieved from the FROG measurement (dashed black line) is compared with the measured autocorrelation profile (red line). (d) The pulse spectrum retrieved from the FROG measurement (dashed black line) is compared with the optical spectrum analyser (OSA) experimental spectrum (red line). (e, f) Temporal and spectral intensity evolution obtained from numerical simulations for a single pulse propagating through 1 km of HNLF. Input conditions are those shown above (panel b), assuming an average input power of 5 mW. Note that both intensity maps are plotted on a logarithmic scale for visual clarity. (g) Spectral supercontinuum intensity as a function of input power, obtained from numerical simulations considering a single pulse propagating into the HNLF. The variations in the simulated supercontinuum spectra illustrate how multiple solitons (and associated dispersive waves) are radiated from the input pulse when increasing the input power.

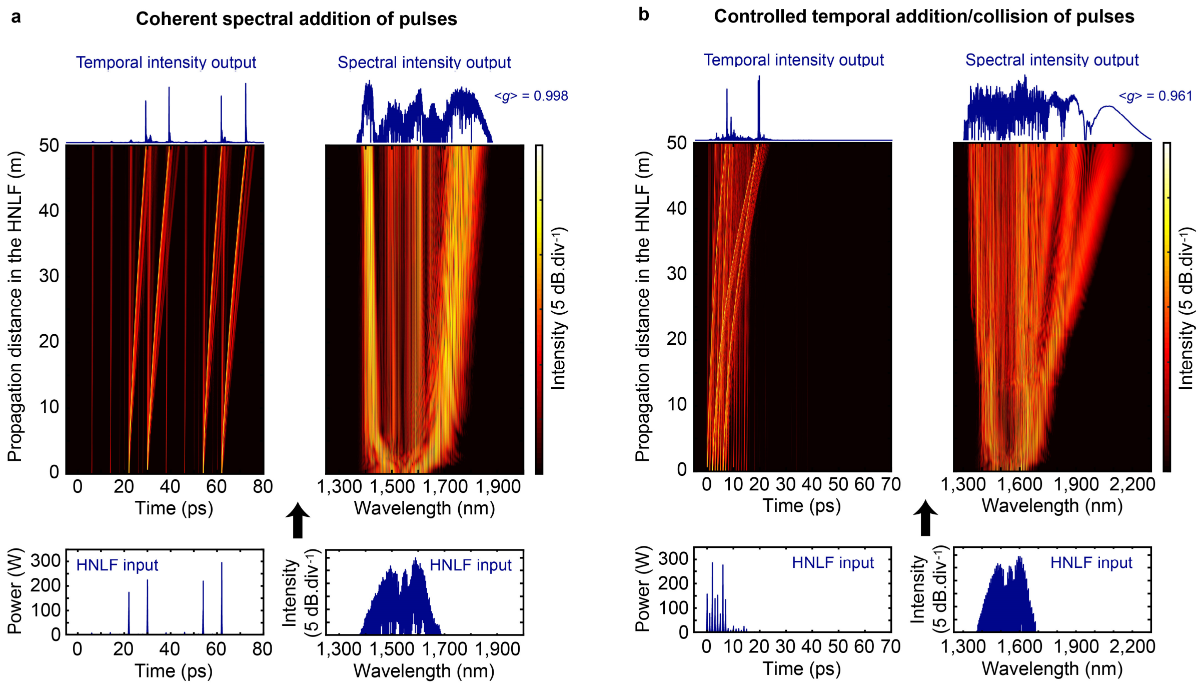

Supplementary Figure 2: Example of supercontinuum generation with extended spectro-temporal control. Two different sets of femtosecond pulse patterns are generated numerically according to the evolution of a single pulse (200 fs Gaussian shape with 2 kW peak power) in the integrated pulse-splitter. After injection of the pulses into the highly nonlinear fibre (HNLF), solitons are ejected and experience different Raman self-frequency shifts, leading to spectral broadening and resulting in the SC properties shown on the top panel. Their respective spectro-temporal evolution in 50 m of HNLF, numerically simulated, illustrate how drastically different the propagation dynamics can be, resulting in very different supercontinuum output properties: In (a), multiple pulses are spectrally superposed in a coherent manner. In (b), two pulses at different wavelengths are temporally colliding.

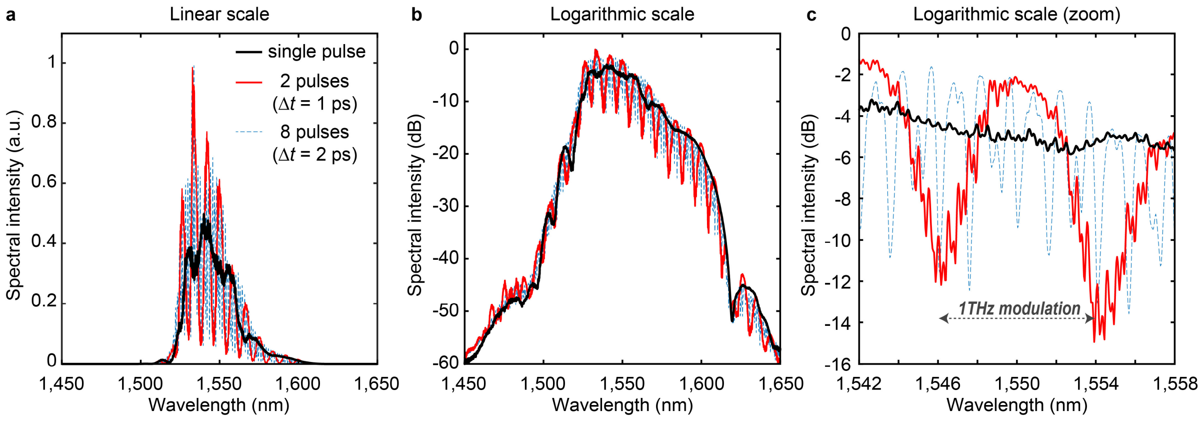

Supplementary Figure 3: Example of spectra measured experimentally at the HNLF input for different pulse pattern configurations. The linear (a) and logarithmic (b) spectra measured experimentally are shown for selected pulse patterns configurations (see legend). The corresponding zoom into the logarithmic spectra (c) shows clear modulation structures of the spectral envelope depending on the number and separation of pulses in the selected pattern.

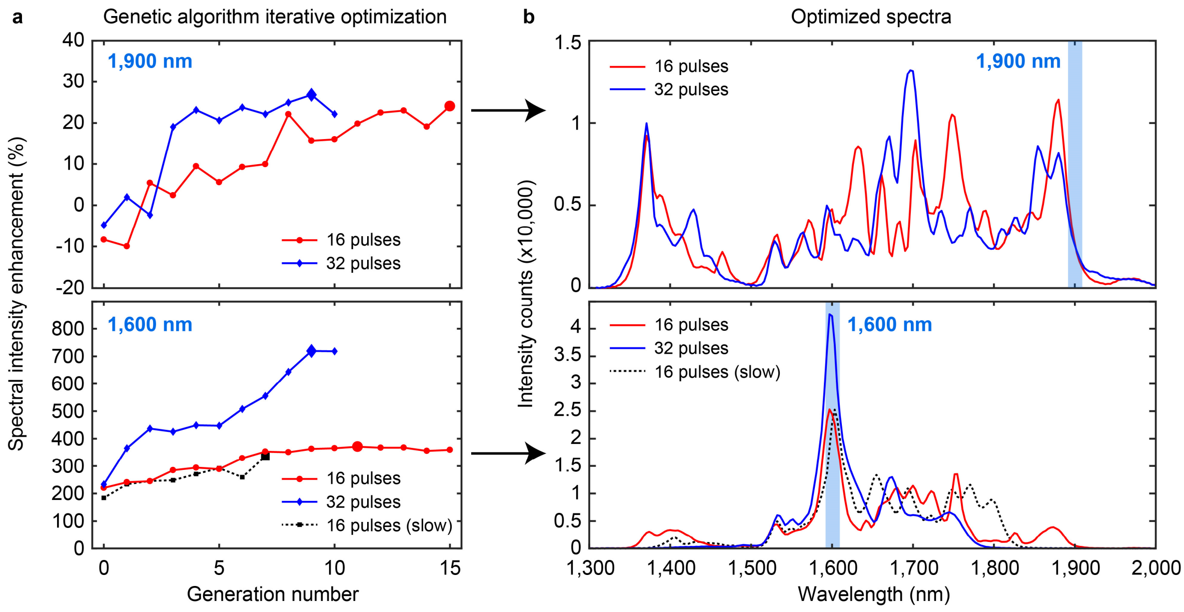

Supplementary Figure 4: Genetic algorithm evolution for various conditions. The evolution of the spectral intensity enhancement (a) and corresponding optimal spectra (b) are shown for an optimization process with a target wavelength at 1900 nm and 1600 nm (see respective blue shadings). The red line shows genetic algorithm (GA) optimization when 5 interferometers were used in the sample (i.e. for 16 pulses) and their splitting ratio actively adjusted for 15 generations of 500 individuals and a settling time of 500 ms. Similar results are reported when 6 interferometers were used in the sample (i.e. for 32 pulses) and their splitting ratio actively adjusted for 10 generations of 1,000 individuals and a settling time of 500 ms (blue line). An example of GA optimization considering a longer settling time (3 s) based on the use of 5 interferometers (i.e. for 16 pulses) and adjusted for 7 generations of 500 individuals is also shown for comparison (black dashed line). The best results in (a), for which the corresponding spectra have been extracted, are highlighted with bold markers.

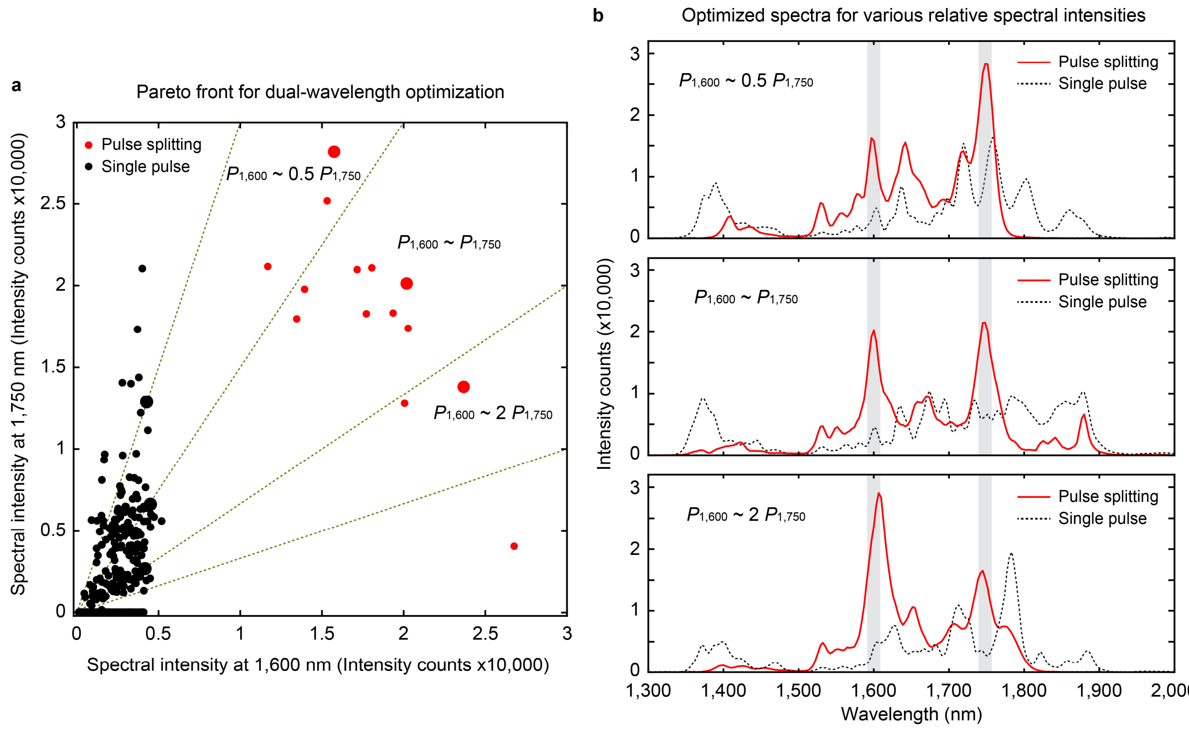

Supplementary Figure 5: Experimental results of dual-wavelength intensity optimization-based genetic algorithm. (a) Experimental Pareto front showing the optimal set of spectral intensities at 1600 nm and 1750 nm retrieved by using a 16 pulse splitting optimization (red dots) and compared with the optimal cases measured using a single input pulse with variable power (black dots). The scatter plot displayed in (a) was separated into three domains corresponding to different relative intensities between the two optimized wavelengths (see domain boundaries illustrated as dashed black lines). For each domain, the best result from the genetic algorithm optimization obtained by analysis of the Pareto front, as well as from a single input pulse scenario, have been extracted (highlighted with bold markers). The corresponding spectra obtained for each case are shown in (b), where the grey shadings correspond to the optimized wavelengths.

Supplementary Discussion

Numerical simulation of supercontinuum generation dynamics for single pulse evolution: In Supplementary Figure 1, we provide an overview of the initial conditions and evolution dynamics of a single pulse propagating into our 1 km-length highly nonlinear fibre (HNLF) for given experimental parameters. The initial pulse properties, measured experimentally at the HNLF input[1], are summarized in Supplementary Fig. 1a-d. Here, the pulse had an initial average power of ~15 mW and was propagated through the chip in the absence of pulse-splitting (i.e. taking the shortest possible path in the sample waveguide). It was then characterized after undergoing amplification in the erbium-doped fibre amplifier (EDFA), in order to reach an average power of up to 50 mW (corresponding to a peak power of ~ 1 kW). The pulse evolution for supercontinuum generation, numerically obtained assuming a 5 mW average input power, is shown in Supplementary Fig. 1e,f. This corresponds to the typical dynamics of coherent supercontinuum generation in a slightly anomalous dispersion regime - where a sub-picosecond input pulse undergoes soliton fission and subsequent Raman self-frequency shift, accompanied by the radiation of dispersive waves, ultimately leading to the formation of a broadband output spectrum[2]. Similarly, in Supplementary Fig. 1g, we show how the supercontinuum spectral output evolves depending on the input pulse power (considering the amplification range used experimentally, i.e. an average power from 0.1 to 50 mW).

These results were obtained via numerical simulations (see Methods), based on our experimental conditions (taking into account the frequency dependence of the fibre attenuation), and considering the single pulse shown in Supplementary Fig. 1b (propagating into our HNLF). The initial pulse had a 200 fs duration with a slight chirp and a dual-peak pulse shape. Its peak power was tuned within the range [2-1000] W (i.e. similar to the experimental conditions reported in Fig. 3a of the main manuscript). We also considered the amplification process and amplified spontaneous emission (ASE) introduced by the EDFA, while including the higher losses of the HNLF outside the spectral window (about 1350 – 1900 nm) to explain the limited spectral bandwidth of the supercontinuum observed experimentally.

From Supplementary Fig. 1, one can clearly observe that there is good qualitative agreement with the experimental results reported in Figure 3 of the main manuscript. As we increase the power, more solitons (and corresponding dispersive waves) are radiated to contribute to the spectral broadening during propagation[3-5] – Note that in our case, spectral broadening induced by soliton self-frequency shift is limited by the significantly high losses above 1900 nm, which cannot be neglected for such a long fibre. Interestingly, although a careful characterization of the experimental parameters has been conducted, it is worth mentioning that some quantitative discrepancies with Figure 3a are observed in our simulation results. This can be attributed to uncertainty in both the fibre and input pulse parameters (e.g. due to an imperfect reconstruction of the input pulse caused by a limited spectral resolution of our frequency-resolved optical gating traces), as well as ambiguity in the type and magnitude of noise (and

distortions) induced by the EDFA for different amplification levels. Additional contributions, such as polarization-mode dispersion and/or nonlinear effects (e.g. cross-phase modulation) due to polarization cross-talk in the integrated waveguides, can also be responsible for discrepancies. The fact that such numerical simulation approaches have been shown to usually provide trustworthy agreement with experimental results[2] is here a key point to highlight the importance of the results reported here: In many cases, several experimental parameters may not be perfectly known, and they are thus difficult to assess *a priori* for modelling a target application. Our approach, enabling the efficient tuning of multiple variables in the parameter space, hence represents a powerful alternative for the optimization of supercontinua - as well as similar complex dynamics observed in a variety of ultrafast nonlinear systems.

For instance, Supplementary Figure 2 presents simulation results illustrating the temporal and spectral evolution of multiple femtosecond pulses sent into 50 m of HNLF. Both panels (a) and (b) show simulation results obtained using the simplified experimental setup and parameters described in the main manuscript (see Fig. 5a). Here, the 64 pulses are generated from the propagation in our integrated pulse-splitter with different (random) splitting ratios (thus yielding different input pulse parameters – see bottom panels). As can be seen in these two simulations, different propagation scenarios can be reached from the same initial pulse, after being prepared in our integrated pulse-splitter. In the first case, coherent spectral superposition is obtained via the generation of multiple pulses at the same wavelength, while in the other case, one can engineer the collision (i.e. temporal superposition) of pulses at different wavelengths[4,6-8]. These numerical results clearly illustrate how this system can enable the control of light emission and nonlinear frequency conversion in an extremely versatile way, where both the spectral and temporal properties of the light source could potentially be controlled in a coherent and reproducible fashion.

<u>Initial input spectra for different pulse patterns:</u> For completeness, we also provide the spectra measured in our experimental setup at the input of the HNLF. The spectra, shown in Supplementary Figure 3 after pulse splitting and amplification, are provided for selected pattern configurations, also depicted in Figure 2 of the main manuscript (in the temporal domain). Starting from the very typical and smooth spectrum, the pulse-splitter leads to coherent temporal modulation, which yields interference in the spectrum. In particular, depending on the integrated pulse-splitter configuration, the otherwise smooth initial spectral envelope (black line) presents a modulation based on the number and separation of the generated pulses. This modulation is relatively clean when considering only the generation of two pulses (see, e.g., the plain red line, for 2 pulses separated by 1 ps) and typically exhibits a contrast above 10 dB (in agreement with the measured extinction ratio of the splitter). As expected, a more complex interference pattern can be observed in the spectrum when considering more pulses (see e.g. dashed blue line, for 8 pulses separated by 2 ps) and therefore, multiple modulation frequencies between the pulses of adjacent patterns.

Genetic algorithm - Optimization approach, results and processing: The evolutionary (genetic) algorithm settings were chosen to provide a suitable supercontinuum in a reasonable time frame. In particular, we observed that even when fully adjusting 5 interferometers' settings in the sample (i.e. changing the pulse path from 0 to 100 %), the predominant spectral modifications were stable and reproducible after only a few hundred ms settling time (including the response time of the electronic driver and spectrometer). In this 'worst-case scenario' of supercontinuum tuning, we found that a perfectly stable and reproducible spectrum was typically obtained after 3 s, when slight thermal cross-talk between adjacent waveguide heaters reached equilibrium.

Based on these observations, we performed several rounds of supercontinuum optimization and found that consistent results could be obtained considering a settling time of 500 ms. Supplementary Figure 4 illustrates typical evolutions of the genetic algorithm (GA) iterative process for different parameters and target wavelengths. In the first example (i.e. with a target wavelength set to 1900 nm), one can see that similar optimization results are obtained regardless of the number of interferometers used. This is due to the fact that, at such a wavelength, a soliton with a minimal power needs to be ejected during HNLF propagation of the input pulses. In this case, the potential spectral optimization is therefore strongly limited (~25%) by the overall power budget of the initial pulse patterns, as well as from the increasing fibre attenuation at wavelengths approaching 2000 nm. In the second example, however, the spectral intensity enhancement obtained at 1600 nm is already significant (~ 370 %) and reached after only a few generations, even when just 16 pulses are used for optimization. At this wavelength, we can observe that an approximately two-fold spectral intensity enhancement (~ 750 %) can be readily obtained using an optimization based on 32 pulses. This confirms that spectral components with a wavelength closer to the optical pump require much less energy to be produced from the initial pump pulse. Increasing the number of pulses enables to drastically enhance the power spectral density at this wavelength via controlled soliton ejection from each pulse (i.e. in a way similar to using a pump laser with increased repetition rate).

For this wavelength, we also performed a GA optimization using longer settling times between various iterations of the integrated splitter thermal adjustments. In Supplementary Fig. 4, we also provide an example of such an optimization procedure with a 3 s settling time, clearly illustrating that the previous optimization results are consistent (~ 350 % for 16 pulses). Note that comparable results were also observed for other wavelength optimization procedures and/or variable settling times (in the range 100 ms – 20 s).

Finally, we mention in the main text that further tunability in the supercontinuum could be obtained from multiple pulse seeding compared to the single pulse case. Besides the ability to control both temporal and spectral location of the radiated structures (see Fig. 5 of the main manuscript), an example of our approach's versatility is illustrated in the dual-wavelength optimization shown in Supplementary

Figure 5. Indeed, in this figure, we only present the optimized supercontinuum, where the two target wavelengths possess similar intensities (i.e. for which the intensity at one wavelength is no more than twice the intensity at the other wavelength). Yet, the optimization of multiple objectives clearly reveals the potential of our pulse splitting approach, allowing to further control the intensity at each wavelength of the supercontinuum. This aspect is illustrated in Supplementary Fig. 5a, where we show the optimization results in the form of a Pareto front[9-11]. In this case, the weight of each optimization criterion (i.e. the spectral intensity for the two wavelengths) can be post-selected to adjust the respective intensity at each optimized wavelength. Typical spectra obtained for dual-wavelength optimization with various relative spectral intensities are reported in Supplementary Fig. 5b.

Once again, one can readily observe that the control of the spectral intensity at both wavelengths of interest is not easily addressed when considering only a single input pulse with adjustable power (as seen by the highly skewed distribution of black dots in Supplementary Fig. 5). On the other hand, a clear intensity enhancement, along with a selective control of the power distribution between the two wavelengths of interest, can be readily achieved using our optimization technique. Such results further highlight the versatility of the proposed pulse splitting scheme as an effective tool for the control of supercontinuum generation and complex (nonlinear) optical processes.

Supplementary References

1. Trebino, R. *Frequency-Resolved Optical Gating: The Measurement of Ultrashort Laser Pulses*. (Springer Science & Business Media, 2012).
2. Dudley, J. M. & Taylor, J. R. *Supercontinuum Generation in Optical Fibers*. (Cambridge University Press, 2010).
3. Gordon, J. P. Theory of the soliton self-frequency shift. *Opt. Lett.* **11**, 662-664 (1986).
4. Kivshar, Y. S. & Agrawal, G. *Optical Solitons: From Fibers to Photonic Crystals*. (Academic Press, 2003).
5. Dudley, J. M., Genty, G. & Coen, S. Supercontinuum generation in photonic crystal fiber. *Rev. Mod. Phys.* **78**, 1135-1184 (2006).
6. Weiner, A. *Ultrafast Optics*. Vol. 72 (John Wiley & Sons, 2011).
7. Andresen, E. R., Dudley, J. M., Oron, D., Finot, C. & Rigneault, H. Nonlinear pulse shaping by coherent addition of multiple redshifted solitons. *J. Opt. Soc. Am. B* **28**, 1716-1723 (2011).
8. Andresen, E. R. & Rigneault, H. Soliton dynamics in photonic-crystal fibers for coherent Raman microspectroscopy and microscopy. *Opt. Fiber Technol.* **18**, 379-387 (2012).
9. Goldberg, D. E. & Holland, J. H. Genetic algorithms and machine learning. *Mach. Learn.* **3**, 95-99 (1988).
10. Coello, C. A. C., Lamont, G. B. & Van Veldhuizen, D. A. *Evolutionary Algorithms for Solving Multi-objective Problems*. Vol. 5 (Springer, 2007).
11. Dasgupta, D. & Michalewicz, Z. *Evolutionary Algorithms in Engineering Applications*. (Springer Science & Business Media, 2013).
